# Causes of stillbirths at Kgapane hospital, Limpopo province

**DOI:** 10.4102/safp.v66i1.5863

**Published:** 2024-04-17

**Authors:** Gert J.O. Marincowitz, Clara Marincowitz

**Affiliations:** 1 Department of Family Medicine, Faculty of Health Sciences, University of Limpopo, Mankweng, South Africa; 2 Department of Psychiatry, Faculty of Health Sciences, Stellenbosch University, Cape Town, South Africa; 3 SA Medical Research Council, Cape Town, South Africa

**Keywords:** stillbirths, perinatal, audit, rural heath, district hospital

## Abstract

**Background:**

Stillbirths are a global public health challenge, predominantly affecting low- and middle-income countries. The causes of most stillbirths are preventable.

**Objectives:**

this study reviewed perinatal clinical audit data from Kgapane Hospital over a 4-year period with a special focus on the factors associated with stillbirths.

**Methods:**

File audits were done for all stillbirths occurring at Kgapane Hospital and its catchment area from 2018 to 2021. The data from these audits were analysed to identify factors associated with stillbirths.

**Results:**

A total of 392 stillbirths occurred during the study period at Kgapane Hospital and its surrounding clinics, resulting in a stillborn rate of 19.06/1000 births. Of the 392 stillbirths recorded, audits were conducted on 354 of the maternal case records. The five most common causes of stillbirths identified were: hypertensive disorders in pregnancy (HDP) (29.7%), intrauterine growth restriction without HDP (11.6%), birth asphyxia (7.1%), premature labour (< 1000 g) (6.5%) and maternal infections (5.9%) including HIV with unsuppressed VL, intrauterine infection, coronavirus disease (COVID) and syphilis. Modifiable factors that can form the basis of improvement strategies should include training, timeous referral, plus improved resources and staffing.

**Conclusion:**

Understanding the causes of stillbirths can guide improvement strategies to reduce this heart-breaking complication of pregnancy.

**Contribution:**

Family physicians working in rural hospitals are also responsible for perinatal care. Understanding the factors associated with stillbirths will guide them to develop improvement strategies to reduce these preventable deaths.

## Introduction

Stillbirths are a global public health challenge predominantly affecting low- and middle-income countries.^1^ Globally, about 2.0 million stillbirths occur each year, and most of these (55%) are from sub-Saharan Africa.^2,3,4^ In South Africa (SA), an upper middle-income country, about two-thirds of perinatal deaths are stillbirths, and approximately 7500 occur every year.^5,6^ Unfortunately, the causes of many stillbirths remain unknown, and unexplained stillbirth accounts for about 25% – 30% of perinatal deaths.^3,5,7^ Of these, the majority of the pregnancies were regarded as healthy at the time of the foetal demise and had not been referred for advanced care.^3^

In 2019, stillbirth rates around the world ranged from 1.4 to 32.2 stillbirths per 1000 total births, with the highest stillbirth rate and the greatest number of stillbirths seen in sub-Saharan Africa, followed by Southern Asia. Globally, 2.0m stillbirths at 28 weeks of gestation or more occur each year resulting in a stillbirth rate of 13.9 stillbirths per 1000 total births.^1,2,3^

In the last two decades, the world reduced the number of stillbirths by almost a third, but sub-Saharan Africa did not show this declining trend – about 0.8m babies have been stillborn in this region every year. In 2000, sub-Saharan Africa accounted for 27% of the global number of stillbirths,^1,2,3^ and this proportion increased to 42% in 2019.^1,2,3^ In sub-Saharan Africa, the number of stillbirths is rising, increasing from 0.77m in 2000 to 0.82m in 2019. This increase has been ascribed to the growth in total births outpacing the decline in the region’s stillbirth rate.^1^

Common causes of stillbirth include intrapartum complications (including hypoxia), antepartum haemorrhage (including placental abruptio), infections and maternal conditions such as hypertensive disorders of pregnancy, with foetal growth restriction as a common underlying pathway.^8^ Many of these causes can be avoided through better nutrition, antenatal care, foetal growth monitoring and access to safe and quality labour care.^3,4,9,10^ Age and smoking can also increase the risk of maternal disease and stillbirth. A United Kingdom (UK) population-based study found that mothers who smoked were nearly four times more likely to experience foetal growth restriction compared to mothers who did not.^1^ Some stillbirths are associated with congenital anomalies and often include neural tube defects, which are preventable with folic acid intake.^1^

One of the most recorded causes of stillbirth in both low- and high-income settings is ‘unknown’ or ‘unspecified’. The inability to establish a cause is a source of great distress for families and hampers the grieving process.^7^ Improving reporting of foetal deaths and perinatal audits could improve the cause of death data quality and availability.^1,2,4^ This would not only help in addressing future preventable stillbirths but could also provide answers for grieving families. We therefore aimed to identify the causes of stillbirths and modifiable factors from audit documents from Kgapane Hospital in the Limpopo province of SA. Findings were compared with the literature, specially focussing on unexplained stillbirths. We used the proportion of unexplained stillbirths as an indicator of the quality of our audits, considering one of the objectives of conducting mortality audits is to establish the cause of death.

## Research methods and design

Kgapane hospital is a medium-sized district hospital (178 beds) in the Mopani District of Limpopo province. It serves a population of 230 000 people and has 21 clinics in its catchment area. Kgapane hospital has a maternity section delivering around 5000 babies annually with 250 or more deliveries done at the 21 clinics in its service area. Monthly, there are 16 fulltime midwives allocated to the maternity section, including the labour ward, ante- and postnatal wards. This amounts to a ratio of one midwife to 3.5 births.^11^

A retrospective observational study was conducted using information from audits conducted on all perinatal deaths occurring in Kgapane Hospital’s catchment area from February 2018 to October 2021, a period during which one of the authors worked at the hospital and conducted monthly audits. Only stillbirths, defined as a foetus born without any signs of life, weighing 500 g or more, were included in this study. Stillbirths where the maternal case records were not available for audit were excluded.

The data-extraction tool that was developed to extract the information from the audit documents is based on the Perinatal Problem Identification Programme (PPIP) data-collection sheet, which is a standardised and tested tool in SA.^6^ All the maternal demographic, obstetric and health data, pregnancy outcomes foetal and neonatal factor plus the factors contributing to the deaths were extracted from the audit reports and captured by the researcher on a password-protected Excel^®^ spreadsheet.

The entered data were checked, cleaned and subsequently analysed descriptively. The demographic characteristics of the mothers and foetuses and the factors contributing to stillbirths were identified and presented as frequencies and percentages.

### Ethical considerations

Ethical clearance for the research was obtained from the Research and Ethics Committee of the University of Limpopo (TREC/61/2023: IR) and permission was obtained from the Limpopo provincial Department of Health (LP 2023-03-012) as well as Kgapane Hospital management. A waiver of consent was obtained from the CEO of Kgapane Hospital because secondary data were used for the study with no implications for the patients. To ensure confidentiality, only patient identification codes were used when capturing the data.

## Results

A total of 392 stillbirths occurred during the period 01 February 2018 to 31 October 2021 at Kgapane Hospital and its surrounding clinics. The total number of births for this period was 20 562, resulting in a stillborn rate of 19.06/1000 births. Of the 392 stillbirths recorded, audits were conducted on 354 of the maternal case records. For the remaining 38 (9.7%), case records were missing and thus not audited.

The demographic information of women who had stillbirths is presented in detail in Table 1. Most women were between 30 and 40 years of age (40%) with a mean age of 27.6 years. Women who never had a viable pregnancy formed the largest subgroup with 34%. This includes women who have previously had miscarriages or stillbirths. Most women (86.4%) delivered their stillbirths in the hospital with 7.6% at clinics, 4% at home and 2% in transit. Multiple pregnancies constituted 7.6% of the stillbirths. Fifteen point five per cent were documented as having had previous miscarriages, and only 1% had had previous stillbirths.

**TABLE 1:** Demographic data of women who had stillbirths (*N* = 354).

Demographic variable	*n*	%	Gravidity		Parity		Yes		No		Unknown	
			*n*	%	*n*	%	*n*	%	*n*	%	*n*	%
**Age**												
< 20 years	42	11.9	-	-	-	-	-	-	-	-	-	-
20–30 years	138	40	-	-	-	-	-	-	-	-	-	-
30–40 years	93	26.2	-	-	-	-	-	-	-	-	-	-
> 40 years	15	4.2	-	-	-	-	-	-	-	-	-	-
Mean	276	276	-	-	-	-	-	-	-	-	-	-
Median	27	27	-	-	-	-	-	-	-	-	-	-
Unknown	66	18.6	-	-	-	-	-	-	-	-	-	-
**Gravidity/Parity**												
0	-	-	NA	NA	120	34	-	-	-	-	-	-
1	-	-	94	26.6	83	23.4	-	-	-	-	-	-
2	-	-	98	27.7	81	22.9	-	-	-	-	-	-
3	-	-	75	21.2	44	12.4	-	-	-	-	-	-
4	-	-	51	14.4	16	4.5	-	-	-	-	-	-
5	-	-	20	5.6	4	1.1	-	-	-	-	-	-
> 5	-	-	10	2.8	0	0	-	-	-	-	-	-
Unknown	-	-	6	1.7	6	1.7	-	-	-	-	-	-
**Risk factors**												
Previous miscarriage	-	-	-	-	-	-	55	15.5	292	82.5	4	1.1
Previous stillbirth	-	-	-	-	-	-	3	0.8	347	98	4	1.1
Previous NND	-	-	-	-	-	-	3	0.8	347	98	4	1.1
Previous infant death	-	-	-	-	-	-	16	4.5	334	94.4	4	1.1
Multiple pregnancy	-	-	-	-	-	-	27	7.6	322	91	5	1.4
**ANC attended at least once**												
BANC	318	89.8	-	-	-	-	-	-	-	-	-	-
Never	29	8.2	-	-	-	-	-	-	-	-	-	-
HRC	126	35.6	-	-	-	-	-	-	-	-	-	-
Unknown	7	2	-	-	-	-	-	-	-	-	-	-
**Place of delivery**												
Hospital	306	86.4	-	-	-	-	-	-	-	-	-	-
Clinic	27	7.6	-	-	-	-	-	-	-	-	-	-
Home	14	4	-	-	-	-	-	-	-	-	-	-
In transit	7	2	-	-	-	-	-	-	-	-	-	-

ANC, antenatal care; BANC, basic antenatal care; HRC, High Risk Clinic; NND, neonatal death.

A total 89.8% of the women attended basic antenatal care at least once, and 35.6% of them also attended the high-risk clinic at the hospital.

**FIGURE 1:**
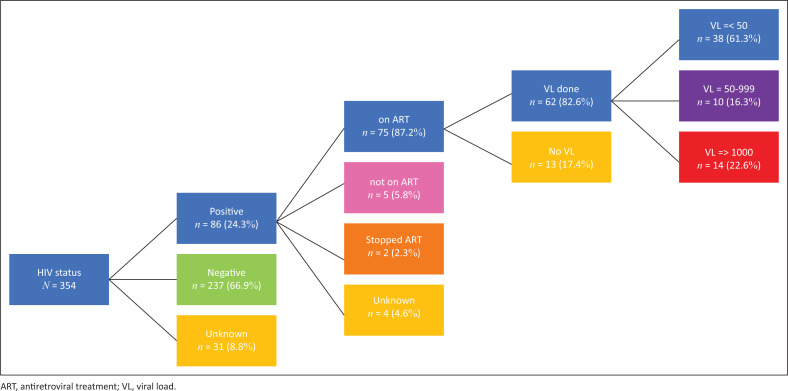
HIV data for women with stillbirths.

The HIV status of 24.3% of the women was positive (in 8.8% the HIV status was unknown). Of those positive, 87.2% were on treatment. Of those on treatment with known viral loads (*n* = 62), 38 (61.3%) had a viral load of less than 50 copies/mL, for 10 (16.3%) the viral load was between 50 and 999 and 14 (22.6%) had viral loads of 1000 and above (Figure 1).

Macerated stillbirths (MSB) constituted 67.2% of the total and fresh stillbirths (FSB) constituted 32.8%. The birth weights of stillbirths are presented in Table 2.

**TABLE 2:** Birth weight of stillbirths (*N* = 354).

Birth weight	All stillbirths (*N* = 354)		MSB (*n* = 238, 67.2%)		FSB (*n* = 116, 32.8%)	
	*n*	%	*n*	%	*n*	%
500 g – 999 g	87	24.6	62	26.1	25	25.6
1000 g – 1999 g	125	35	91	38.2	33	28.4
2000 g – 2999 g	82	23.2	54	22.7	28	24.1
3000 g – 3999 g	48	13.6	26	10.9	22	19
> 4000 g	7	2	2	0.8	5	4.3
Unknown	6	1.7	3	1.3	3	2.6

FSB, fresh stillbirths; MSB, macerated stillbirths.

**FIGURE 2:**
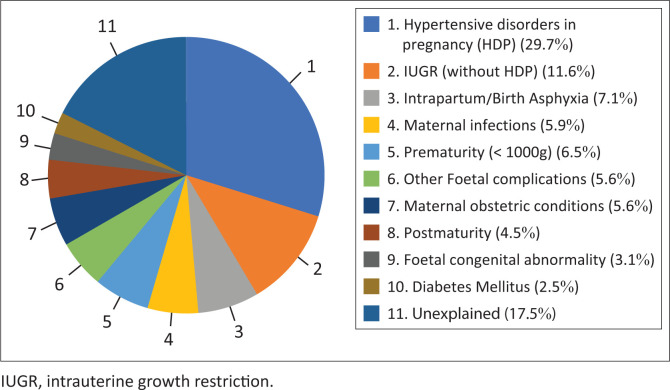
Causes of stillbirths including both macerated stillbirths and fresh stillbirths.

**FIGURE 3:**
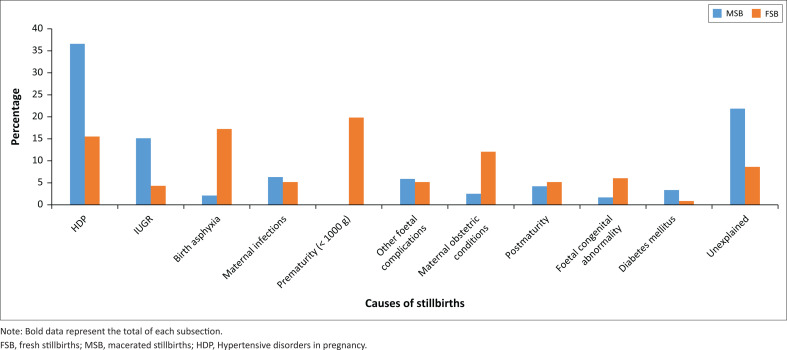
Causes of macerated stillbirths and fresh stillbirths.

The five most common factors identified as contributing to stillbirths were: hypertensive disorders in pregnancy (HDP) (29.7%); intrauterine growth restriction without HDP (11.6%); birth asphyxia (7.1%); premature labour (< 1000 g) (6.5%) and maternal infections (5.9%) including HIV with unsuppressed VL, intrauterine infection, coronavirus disease (COVID) and syphilis. Unexplained stillbirths constituted 17.5% (see Table 3 and Figure 2). Of interest is the difference in contributing factors between MSB and FSB (Figure 3). Hypertensive disorders in pregnancy, IUGR and unexplained were more prevalent in MSB, and asphyxia and prematurity were more prevalent in FSB.

**TABLE 3:** Causes of stillbirths.

Causes of stillbirths	All (*N* = 354)		MSB (*n* = 238)		FSB (*n* = 116)	
	*n*	%	*n*	%	*n*	%
**Hypertensive disorders in pregnancy (HDP)**	**105**	**29.7**	**87**	**36.6**	**18**	**15.5**
PET	44	-	35	-	9	-
Gestational HT	42	-	34	-	8	-
Chronic HT	14	-	14	-	0	-
HELLP syndrome	5	-	4	-	1	-
**IUGR (without HDP)**	**41**	**11.6**	**36**	**15.1**	**5**	**4.3**
**Intrapartum/Birth Asphyxia**	**25**	**7.1**	**5**	**2.1**	**20**	**17.2**
**Maternal infections**	**21**	**5.9**	**15**	**6.3**	**6**	**5.2**
HIV with unsuppressed VL	14	-	9	-	5	-
Intrauterine infection	5	-	4	-	1	-
COVID	1	-	1	-	0	-
Syphilis	1	-	1	-	0	-
**Prematurity (< 1000 g)**	**23**	**6.5**	**0**	**-**	**23**	**19.8**
**Other foetal complications**	**20**	**5.6**	**14**	**5.9**	**6**	**5.2**
Cord around the neck	10	-	8	-	2	-
Discordant growth in two sets of twins	5	-	5	-	0	-
Cord prolapse	3	-	0	-	3	-
Calcified placenta @ 36/40	1	-	1	-	0	-
Breech	1	-	0	-	1	-
**Maternal obstetric conditions**	**20**	**5.6**	**6**	**2.5**	**14**	**12.1**
Placentae Abruptio	12	-	5	-	7	-
Uterine rupture	6	-	0	-	6	-
Antepartum haemorrhage	2	-	1	-	1	-
**Postmaturity**	**16**	**4.5**	**10**	**4.2**	**6**	**5.2**
**Foetal congenital abnormality**	**11**	**3.1**	**4**	**1.7**	**7**	**6.0**
**Diabetes mellitus**	**9**	**2.5**	**8**	**3.4**	**1**	**0.9**
**Trauma**	**1**	**0.3**	**1**	**0.4**	**0**	**-**
**Unexplained**	**62**	**17.5**	**52**	**21.8**	**10**	**8.6**

**Total**	**354**	**100.0**	**238**	**100.0**	**116**	**100.0**

Note: Bold data represent the total of each subsection.

FSB, fresh stillbirths; MSB, macerated stillbirths; COVID, coronavirus disease; PET, pre-eclamsia; HT, hypertension; IUGR, intrauterine growth restriction; HELLP syndrome, haemolysis, elevated liver enzymes, low platelets.

The four most common modifiable factors identified were: inadequate implementation guidelines, patient-related factors and non-referral to the next level of care. The four guidelines most frequently inadequately followed were the management of HDP, basic antenatal care guidelines including plotting of symphysis fundal height, the early identification and management of intrauterine growth restriction and the management of HIV during pregnancy.

## Discussion

In this study, the five most frequently identified factors contributing to stillbirths at Kgapane hospital were: HDP (29.7%), intrauterine growth restriction without HDP (11.6%), birth asphyxia (7.1%), premature labour (< 1000 g) (6.5%) and maternal infections (5.9%), which included mostly HIV with unsuppressed VL. We were unable to find a contributing factor in 17.5% of cases. In MSBs, unexplained stillbirths accounted for 21.8%, while among FSBs, there were 8.6% without a contributing factor identified. Given the fact that HIV remains a significant cause of stillbirths in sub-Saharan Africa,^1,2,3,6^ our findings that the maternal HIV treatment rate was only 87.2% and the viral load suppression rate was 61.3% are concerning.

The causes of stillbirths identified in our study are very similar to the literature.^3,8,9,10,12,13,14^ Stillbirths were mostly recorded as unexplained intrauterine death (33.1% – 50%), hypertensive disorders (10% – 23%), intrapartum asphyxia (15.6% – 24%), antepartum haemorrhage (4.9% – 16.3%), foetal abnormality (3.5% – 4.4%), intrauterine growth restriction (1% – 5%), maternal disease (2.7%), preterm labour (2.6%) and infections (1.8%).^9,10,12^

The PURPOSe study conducted in Southeast Asia found that the confluence of maternal hypertensive conditions and placental malperfusion leading to growth restriction and asphyxia were a major cause of stillbirths.^8^ Birth weights less than the 3rd percentile were associated with antepartum stillbirth, and the greatest risk was seen in babies not suspected to have been growth-restricted antenatally.^13^

Madhi et al. conducted placental macroscopic and histopathological examination and blood cultures on stillborn foetuses to identify causes of stillbirths, and they identified that a significantly higher number of stillbirths were caused by infections (19% [placental infection in 4% and foetal bacterial infection in 16%]) and pathological placental conditions (19%).^14^

Our finding that unexplained stillbirths accounted for 17.5% of the total stillbirths is very similar to what Madhi et al. reported (18%), while it is less than what is reported in other studies, with a range of 34% – 50%.^5,6,7,9,10,14^ These differences in rates could be attributed to interpretation, differences in research focus or merely chance.

Intrapartum foetal monitoring, poor implementation of key treatment guidelines, patient-related factors and non-referral to specialised levels of care are common modifiable reasons contributing to stillbirths.^5,6,7,8,9,10,12,13,14,15^ Inadequate intrapartum monitoring could be attributed to inadequate staffing and resources. The recommended ratio in low-resource settings by FIGO (The international federation for gynaecologists and obstetricians) is 1.71 births per midwife, and the FIGO ideal ratio would be 1.52.^11^ However, in the maternity section where the present study was conducted, the ratio was 3.5 births per midwife. Focussing training and improvement strategies on the most common causes of stillbirths could assist with improving perinatal outcomes in understaffed healthcare settings.

## Conclusion

Our study described the causes of stillbirths, identified during record audits, at a rural and level-one hospital of which the most significant were HDP, IUGR, intrapartum asphyxia, maternal infections and prematurity. There are several studies describing similar findings regarding perinatal mortality in SA, but these were mainly conducted at referral institutions in urban settings or with the analysis of PPIP data. Of note is the fact that in only 17.5% of stillbirths we could not find any contributing factor or cause. Modifiable factors that can form the basis of improvement strategies should include focussed training, timeous referral, plus improved resources and staffing.

Adequate resources are essential to improve effective intrapartum monitoring.^16^ Maternity ward staffing norms should be standardised.^11^ The inability to establish a cause hampers the grieving process and the ability to prevent stillbirths in future.^7^ The evidence-based guidelines referred to earlier were developed and widely available.^17,18,19^ The importance of perinatal audits lies in the fact that it is widely accepted that knowing factors contributing to perinatal mortality will help to plan improvement.^16^

## Recommendations

Care can be improved by focussing training and monitoring on the implementation of the guidelines for the most common causes of stillbirths. Resources for, and the practice of intrapartum monitoring, as well as the midwife:birth ratio in maternity wards at level-one hospitals must be improved. Referrals of patients who require specialised care need to increase, and an active effort must be made to encourage better health-seeking behaviour among patients, for example, enrolling early for basic antenatal care (BANC), and responding timeously to signs and symptoms that might indicate a risk for the pregnancy.

The most important topics for training are the identification and management of HDP, management of HIV in pregnancy (especially strategies to ensure viral load suppression), management of IUGR and the basic antenatal care guidelines. With the latter the emphasis must be on the correct measurement of symphysis fundal height as a gauge of foetal growth for the early identification of intra-uterine growth restriction at the primary care level and the correct management of IUGR at level-one hospitals.

Audit tools to monitor this will go a long way in strengthening the implementation of these guidelines.^16^ There is a need for further research into staffing norms and its effect on perinatal health outcomes in SA.

### Limitations of the study

Limitations of this study include the fact that retrospective data were used. Often recordkeeping is not at the expected standard. Because the file audits were conducted by the researcher who also worked in the ward by that time, it could compromise the objectivity and introduce bias. Another limitation is that some files were not traced and therefore not audited. Even though the record clerks were requested to look for the missing files several times, 38 files were not traced constituting 9.7% missing data. To claim that the audits have identified the causes of the stillbirths is not correct as there were no autopsies or biopsies conducted. The causes identified can therefore serve as a guide.
